# Laser Therapy in the Treatment of Female Urinary Incontinence and Genitourinary Syndrome of Menopause: An Update

**DOI:** 10.1155/2019/1576359

**Published:** 2019-06-04

**Authors:** Damir Franić, Ivan Fistonić

**Affiliations:** ^1^Outpatient Clinic Ob&Gyn, Rogaška Slatina, Slovenia; ^2^School of Medicine, University of Maribor, Slovenia; ^3^Ob&Gyn and Menopause Clinic, Zagreb, Croatia; ^4^Institute for Women's Health, Zagreb, Croatia

## Abstract

Vaginal birth trauma is the leading cause of stress urinary incontinence (SUI) in women. Also, the process of ageing and hormonal deprivation in postmenopause alters the metabolism of connective tissues and decreases collagen production leading to pelvic floor dysfunction. Noninvasive treatment is recommended as first-line management of urinary incontinence (UI) in women. Surgical procedures are more likely to be implemented to cure UI but are associated with more adverse events. Sex hormone deficiency affects changes also in the lower urinary tract where estrogens are the main regulators of physiological functions of the vagina. In the last decade, laser treatment of SUI and of the genitourinary syndrome of menopause (GSM) has been shown a promising treatment method in peer-reviewed literature. This review's aim is to present the evidence-based medical data and laser treatment of SUI and GSM in an outpatient setting to be a good treatment option, regarding short-term as well as long-term follow-ups. Long-term follow-up studies are needed to confirm that laser treatment is a good, painless outpatient procedure with no side effects in postmenopausal women.

## 1. Introduction

In the female population, conventional treatments of SUI include noninvasive (pelvic floor muscle training), minimally invasive (bulking methods), less invasive (tape and mesh), and invasive surgical procedures. Less invasive operative techniques are related to >15% of complications (bleeding, erosions, urethral injury, infection, chronic pain, and urinary retention) [1], whereas the conventional surgery relates to anesthesia risks and high recurrence rates (25%) [2].

The association between SUI and collagen is well established. The expression levels of Type I and Type III collagen are significantly lower in patients with SUI when compared to the control group (*p*<0.01) [3], as well as pubocervical fascia of incontinent women [4]. Lack of hormonal support in menopause additionally depletes collagen reserve. That is a possible explanation for the failure of many surgical procedures in urogynecology with a frequent recurrence of symptoms [5].

Scientific and technological progress has led to better clinical outcomes with less invasive procedures with shorter recovery times and lower implicated costs. In this sense, recent evidence supports laser treatment as an alternative and effective intervention for SUI [6].

Almost 50% of menopausal women experienced symptoms of the genitourinary syndrome of menopause (GSM) during their lifetime. [7]. More than one-third of postmenopausal women on systemic hormone therapy (HT) express the symptoms of GSM thus needing additional local estrogen therapy. Therefore, laser treatment might improve the symptoms of GSM especially in women where local estrogen therapy is contraindicated (i.e., breast cancer survivors) [7].

## 2. Laser Mode of Action

Pulsed laser photothermal energy can improve collagen structure and initiate neocollagenesis in the skin [8] and pelvic floor with nearby tissue [9]. Elevation in temperature up to 63°C increases the contraction of collagen fibers in vaginal epithelium and provokes neocollagenesis, elastogenesis, neoangiogenesis, and increased fibroblast pool, as well [10]. In addition, morphometry showed an increase in the volume density of blood capillaries and the thickness of the epithelial layer [10].

The Er:YAG laser SMOOTH® (Fotona, Slovenia) is a noninvasive nonablative laser procedure for the functional strengthening of connective tissue inside the vaginal wall, improving the pelvic floor support and diminishing symptoms of pelvic floor dysfunction. Er:YAG laser energy is strongly absorbed in water. Therefore, laser pulses achieve controlled heating of the collagen in the deeper mucosa layers (lamina propria), with no ablation or overheating of the mucosa surface, reducing the risk of perforation or accidental lesions of the urethra, bladder, or rectum. The recommended parameters are as follows: laser spot size of 7 mm, the frequency of 1.6 Hz, and fluence (laser energy emitted per unit area) of 6.0 J/cm^2^. Mechanical pull of the deeper tissue layers following shrinkage of the upper, photothermally processed tissue layers further contributes to the tightening effect [11]. No general anesthesia is needed. The lower vaginal third and introitus can be covered with a thin layer of anesthetic cream. Treatment regime consists of three sessions 3 to 4 weeks apart. When the process of neocollagenesis is well on its way and assuming the patient Collagen Remodeling Capacity (CRC) is not fully reached during the first session, some previously not affected collagen fibers are additionally captured with the second and third session. Minor side effects include a sensation of warmth, increased vaginal discharge, and only rarely transient urge urinary incontinence [12].

CO_2_ laser system MonaLisa Touch® (DEKA, Florence, Italy) is a fractional ablative intravaginal therapy that is delivered once a month for 3 consecutive months. No matter that laser settings could be adjusted according to the age and indication for the procedure, the recommended settings [13, 14] of the microablative fractional CO_2_ laser (MFCO2-Laser) are the following: D-Pulse mode, dot power 40 W; dwell time 1,000 *μ*s; and dot spacing 1,000 *μ*m for the treatment of the vaginal canal and the dot power 24 W; dwell time, 400 *μ*s; and dot spacing 1,000 *μ*m for the treatment of the vaginal introitus. The procedure is usually performed in an outpatient setting and does not require any specific preparation or anesthesia. It is recommended to avoid vaginal sexual intercourse for at least 3 days after the laser application in order to prevent an inflammatory reaction which might last up to 48 h.

## 3. Laser Treatments for Stress Urinary Incontinence

The first pilot study regarding Er:YAG laser in the treatment of female SUI started on September 20^th^, 2009 [15]. The degree of incontinence and its impact on the quality of life (QoL) were assessed with the self-reported International Consultation on Incontinence Questionnaire–Urinary Incontinence Short Form (ICIQ-UI SF) [16], where a maximum score of 21 represented permanent incontinence.

**Figure 1:**
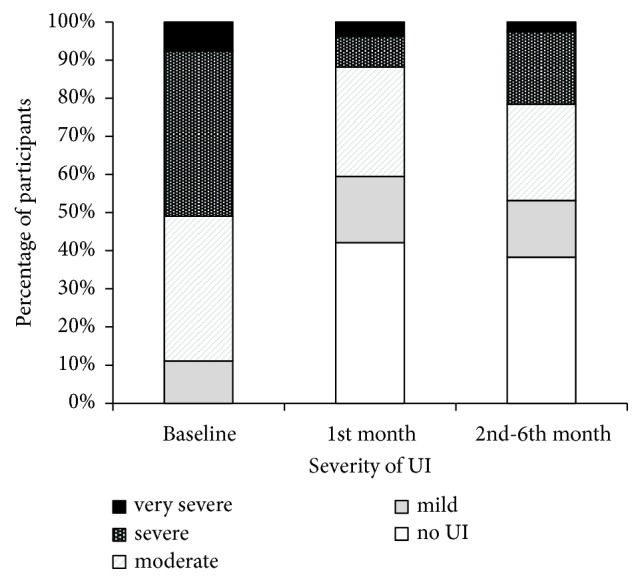
Kloving's categories of ICIQ-UI SF score severity at the baseline and follow-up visits. ICIQ-UI, Consultation on Incontinence Questionnaire-Urinary Incontinence-Short Form; UI, urinary incontinence. Reproduced with permission (Taylor & Francis [15]).

At the first follow-up measurement (1 month after the intervention) the number of completely continent patients increased from zero to 17/52 (42.3% continent). At the second follow-up measurement, 2–6 months after the intervention, 18/47 (38.3% continent) had an ICIQ-UI score = 0 (Figure 1). From baseline to the second follow-up, 34/47 (72.3%) participants experienced improvement, whereas 11/47 (23.4%) experienced no change in the ICIQ-UI score, and two (4.3%) experienced worsening of symptoms. No major adverse events were noticed or reported throughout the course of laser treatment and the follow-up period. The rare mild reported symptoms such as slight edema, vaginal discharge, and transient urgency vanished spontaneously after 8 days.

Ogrinc BU et al. published a study on 175 women with newly diagnosed SUI (66%) and mixed urinary incontinence (MUI, 34%) [17], treated according to the uniform Er:YAG laser protocol. Follow-ups were performed at 2, 6, and 12 months after the procedure. The results were based on the Incontinence Severity Index (ISI) and the reduction in ICIQ-UI SF scores. One year after laser treatment, 77% of the SUI patients and only 34% of the MUI remained continent. No difference in the efficacy was noted between pre- and postmenopausal patients.

In another study [18] using the same Er:YAG protocol for SUI, urodynamic studies, pad testing, lower urinary tract symptoms (LUTS), and sexual function were assessed before and after treatment. The authors concluded that the procedure for mild SUI was effective at 6-month follow-up, but not in the patients with an initial pad weight >10 g. Moreover, it improved LUTS, QoL, and sexual function. Urodynamic values did not differ across the timeline. The authors speculate that this paradox originates from tighter and more elastic collagen that acts as a “hammock,” preventing urine leakage and reducing pad weights. Although the follow-up was only up to the sixth month, the authors summarized that IncontiLase™ should not replace mid-urethral sling (MUS) surgery as the standard therapy for SUI patients who fail to improve following first-line therapy. In addition, the authors stressed that the injection of bulking agents has been reported to have a cure rate of 53–73.2%, which is better than the cure rate of 39.3% they achieved at 6 months with IncontiLase™. In conclusion, the authors admitted that, based on its minimally invasive nature and the lack of significant adverse effects, the IncontiLase™ procedure may be used as an alternative therapy for mild SUI cases.

In a long term, a 24-month follow-up study [19] on 114 postmenopausal women suffering from SUI, the vaginal erbium laser (VEL) treatment induced a significant decrease in baseline ICIQ-SF scores of 12.2 ± 2.5. The ICIQ-UI SF scores remained significantly (p<0.01) lower than the basal values after 1 (4.8 ± 1.8), 3 (6.2 ± 1.9), 6 (7.0 ± 2.3), and 12 (8.0 ± 1.8) months after the last VEL application. However, the scores after 18 (9.3 ± 2.7) and 24 (9.9 ± 2.8) months from the last VEL application were not significantly different from the baseline values.

Several other observational studies in which Er:YAG was used for mild to moderate SUI also showed improvement in SUI symptoms [20–22]. To date, there is only one patient-blinded randomized controlled trial for SUI [23] consisting of 114 women receiving a single session of nonablative thermal-only Er:YAG laser treatment. This study reports improvement of SUI symptoms, QoL (ICIQ-UI SF), and sexual function (PISQ12 and FSFI) in premenopausal parous women. A 21.4% (12/56) of the laser group patients were continent 3 months after treatment according to ICIQ-UI SF (score = 0) in comparison to only 3.6% (2/56) continence in the sham control group. The covariates age, BMI, and parity had no significant effect on the outcome. All pelvic floor muscle variables, derived from perineometry studies (duration and maximum pressure), showed a significant improvement in the laser group but not in the sham control group.

Carbon (CO_2_) laser has been used for GSM treatment, particularly focusing on the vulvovaginal atrophy segment [9]. To date, very few studies regarding CO_2_ laser treatment of SUI have been published.

Isaza et al. [24] used the SmartXide2 V2LR fractional microablative CO_2_ laser system (MonaLisaTouch™; DEKA, Florence, Italy) in a prospective study on 161 postmenopausal women suffering from mild SUI. The patients received four sessions 30–45 days apart. SUI was evaluated using the 1-h pad test and the ICIQ-UI SF at baseline and at 12, 24, and 36 months. The basal ICIQ-UI SF score (14.34 ± 2.65) significantly decreased at 12 (7.09 ± 1.1, p < 0.001), 24 (7.49 ± 0.94, p < 0.001), and 36 months (6.76 ± 0.82, p < 0.001) of follow-up. The 1-hour pad test reduced from 9.89 ± 0.57 g at baseline to 3.52 ± 1.89 g, 3.55 ± 1.88 g, and 3.72 ± 2.05 g at 12, 24, and 36 months, respectively (all p < 0.001). Histology analysis which was done before and 6 weeks after the first treatment showed essentially thicker epithelium with a higher population of intermediate and superficial cell shedding. CO2 laser ablative vaginal treatments could increase the possibility of vaginal scarring and infection. Using the nonablative laser treatment, possible side effects could be reduced [23].

The significant improvement in dyspareunia, dryness, burning, itching, dysuria, urgency, and SUI scores was evaluated in the prospective observational study including postmenopausal women with moderate to severe clinical signs of GSM [25]. The women received intravaginal therapy with CO_2_ laser system (MonaLisa Touch®, DEKA, Florence, Italy), once a month for 3 consecutive months. As a secondary outcome, the authors noted that urinary symptoms improved, as the scores of the urinary and QoL questionnaires decreased significantly. ICIQ-UI SF at the baseline was 8.1±5.6 vs. 3.4±4.3 at the 3-month follow-up. All participants showed a > 5-point improvement in the King's Health Questionnaire (KHQ) score, which includes psychometric aspects of UI. The authors concluded that the factors predictive of ideal CO_2_ laser therapy candidates were not identified. Considering predictive, preventive, and personalized medicine (PPPM) the current goal is to predict not only the risk of an adverse clinical event but also the benefits [26]. A recent study [27] identified predictors for the segment of patients achieving optimal Er:YAG laser treatment short-term outcomes. The best results after Er:YAG laser treatment of SUI should be expected in younger women (< 47.5 years) with a body mass index of < 23.3, average newborn birth weight of > 3.6 kg, ICIQ-UI at a baseline of < 10, and a perineometer squeeze duration at a baseline of > 3.51 seconds.

However, despite the rigorous selection of patients, laser treatment will not succeed in a certain group. Namely, SUI is induced by urethral hypermobility not only as a result of weakening or disruption of the pelvic floor musculature and/or pubourethral ligament but also due to the weakening of the urethral sphincter, resulting in more severe intrinsic sphincter deficiency (ISD) [28]. The urethral sphincter function depends on the muscular component, the rhabdosphincter, extending along 60–70% of the urethral length, and the mucosal or intrinsic component, extending across the urethra and contributing to urethral closure [29].

Women whose urodynamic studies showed a maximal urethral closure pressure (UCP) of less than 40 cm H_2_O received three fractional CO_2_ laser treatments four weeks apart in the study of Patel [30]. Post-hoc urodynamic reevaluation, three months after the treatment, showed an increase in maximal UCP of 19-33 cm H_2_O to 45-73 cm H_2_O.

## 4. Overactive Bladder

Perino et al. [31] analyzed the effect of CO_2_ laser treatment in postmenopausal women with overactive bladder (OAB) symptoms (≥ 8 times micturition/24h) and ≥1 symptoms of GSM (itching, burning, reduced lubrication, and superficial and/or severe dyspareunia) in the previous 3 months. OAB symptoms were assessed using the validated Overactive Bladder Questionnaire Short Form (OAB-Q SF). The results at a 1-month follow-up after the 3^rd^ laser session indicated a significant reduction of the number of micturitions and the number of urge episodes (p < 0.0001). Since atrophy of muscles and reduction of collagen content may be important factors in the increased prevalence of UI, the authors stress that fractional CO_2_ laser system can irradiate deeper layers of the vaginal wall, ultimately enhancing tissue tropism and reactivating the extracellular matrix and collagen synthesis, with beneficial effects in the 3 layers of the vaginal wall, in contrast to estrogens or other local therapies that treat the epithelium only.

Besides improvement in SUI episodes using Er:YAG protocol, Tien and coauthors [18] also found a positive effect over OAB, as evidenced by the improvements in Urgency Severity Scale Questionnaire (USS), Overactive Bladder Symptoms Score Questionnaire (OABSS), nocturia episodes, and daytime frequency episodes. Since the majority of women with stress predominant MUI experience significant improvement in OAB symptoms following incontinence surgery [32], the authors speculate that their findings may be at least partly related to SUI improvements following laser treatment.

In patients with SUI, urine leakage into the proximal urethra may stimulate urethral afferents and facilitate the voiding reflex [33]. Lin et al. [34] hypothesized that laser therapy may slightly increase the entire urethral pressure, including proximal urethral pressure, and in turn alleviate OAB symptoms due to a reduction of the bladder reflex response observed in SUI patients. The treatment with Erbium:YAG laser included two sessions. The second session was 4 weeks after the first one. The results concerning OABSS scores were significantly improved after the 3-month follow-up (p < 0.027), with particular impact on urinary frequency (p < 0.001). Unfortunately, OABSS scores were not equal at the 12-month follow-up. By most patients' report, the optimal therapeutic effect was maintained for the duration of three to six months, similar to the results observed by Fistonic et al. [15] (2-6 months). Neocollagenesis induced by Er:YAG SMOOTH® mode can change the composition of the pelvic floor structures and thus increase the pressure over the entire length of the urethra. In SUI patients, the increased proximal urethral pressure may alleviate OAB symptoms by reducing the bladder reflex response.

## 5. Nonablative Photothermal Er:YAG Laser and Microablative Fractional CO_2_ Laser in SUI Treatment: Differences

Lasers used in SUI treatment emit thermal energy at different wavelengths (Er:YAG 2,940 nm; CO_2_ 10,600 nm), but they both induce similar changes related to increased tissue trophysm such as retraction of collagen, neocollagenesis, elastogenesis, the enhanced density of connective particles, and neovascularization. CO_2_ laser thermal action spreads to the depth of 50-125 *μ*m in the vaginal tissue, causing superficial vaporization. Under the same conditions, Er:YAG laser reaches only 5-20 *μ*m in depth with no ablation at all [9].

**Figure 2:**
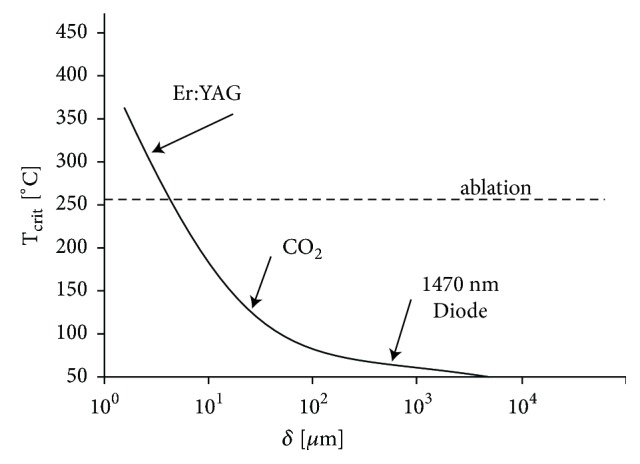
Critical temperature depends on penetration depth. Reproduced with permission (Laser and Health Academy [38]).

Er:YAG laser has 10 to 15 times the affinity for water absorption compared with the CO_2_ laser. Mucous membranes have a very high percentage of water, which is a good target for the Er:YAG laser beam. Because of the extremely high absorption in water, the incident photon energy is almost totally attenuated in the first few micrometers of the tissue, producing, at appropriate parameters, a very controlled column of ablation with an extremely narrow band of secondary coagulation. This process has been known as residual thermal damage (RTD) [35]. This translates into shorter downtime with quicker healing and has been the cornerstone for the use of the Er:YAG in full-face ablative laser resurfacing when compared to the CO_2_ laser, which has a much larger RTD zone [36]. This was the rationale for Lee to use Er:YAG in the treatment of vaginal relaxation syndrome [37]. The author emphasizes that the depth control associated with the Er:YAG wavelength offers major benefits as ablative damage depth is minimized. Multiple micropulses create a shallow, few *μ*m-thick epidermal windows in the vaginal epithelium with minimal RTD, and subsequent micropulses create a pulse-stacking effect without further ablation, but with thermal build-up down into the lamina propria, increasing the RTD zone. Only Er:YAG laser is characterized by the critical temperature above the ablation temperature, making this laser the safest medical laser for Dual Tissue Regeneration mechanism (DRM) nonablative resurfacing (Figure 2) [38].

Atanasiou et al. [14] stressed, based on the studies by Hutchinson-Colas et al. [39], that Er:YAG laser has a thermal effect only, whereas the MFCO_2_-Laser has both ablative and thermal effects, thus stimulating heat shock proteins and other factors (e.g., TGF-*β*), promoting neocollagenesis.

## 6. Vaginal Microbiota

An effect of laser on vaginal microbiota has been reported by Athanasiou et al. [14]. They assessed the effect of microablative fractional CO_2_ laser (MFCO_2_-Laser) therapy on the vaginal microenvironment of postmenopausal women. The findings suggest that, in their sample of 53 postmenopausal women with moderate to severe GSM symptoms, the completion of three laser therapies (at monthly intervals) significantly increased Lactobacillus (p < 0.001) and normal flora (p < 0.001). Those changes consequently decreased vaginal pH from 5.5 ± 0.8 (baseline) to 4.7 ± 0.5 (3^rd^ month, p < 0.001). Therefore, the prevalence of Lactobacillus changed from the baseline value of 30% to 79% at three months. Nevertheless, signs and symptoms of bacterial vaginosis or candidiasis did not appear in the participants included in the study. Although significant decreases were observed only in E. coli and Mobiluncus, there was a trend of lower growth of all Lactobacillus antagonists.

The authors suggest that the observed increase in the normal vaginal epithelial cells confirms the results of the histological study of Zerbinati et al. [40] where it was demonstrated that one of the effects of the MFCO_2_-Laser therapy on the vaginal mucosa was a high degree of epithelial exfoliation, with superficial cells filled with glycogen shedding at the epithelial surface. In conclusion, the authors believed that MFCO_2_-Laser therapy is a promising treatment for the improvement in postmenopausal vaginal health, aiding the repopulation of the vagina with normally existing Lactobacillus species, and reconstituting the normal flora as that observed in the premenopausal status.

## 7. Laser Devices in GSM Therapy

Sex hormone deficiency influences many organ systems including the lower urinary tract. The genital tract is particularly sensitive to a decline in estrogen levels and approximately half of postmenopausal women experience the symptoms of vaginal atrophy that affect sexual function and QoL. The clinical manifestations of vaginal atrophy generally occur 4-5 years after menopause and 25-50% of postmenopausal women present with objective changes as well as with individual symptoms. The most common symptoms of vaginal atrophy include dryness (75%), dyspareunia (38%), burning sensation, discharge, and pain (15%) [41]. Discussing the possibility of CO2 laser to restore the vaginal mucosa for GSM in postmenopausal women, Salvatore et al. [42] investigated microscopic, ultrastructural, and biochemical modifications of the postmenopausal atrophic vaginal mucosa. They made mucosal biopsies (before the treatment and 1 hour after it) to find the real impact of CO2 laser on the vaginal mucosa: the epithelium before the treatment did not present any superficial desquamation, and its basal surface appeared relatively smooth. The connective tissue was stained. One hour after the treatment the epithelium was thicker, and the connective tissue penetrated into the epithelial layer constituting newly formed papillae. Also, many penetrating small vessels were observed inside them. This proved the immediate influence of CO2 laser therapy on vaginal mucosa. The newly formed collagen was also increased in the treatment group from 2.18 to 10.52 nm with a slight decrease in thick fibers (old collagen).

Siliquini GP et al. [7] assessed 87 postmenopausal women before and after the treatment using VAS for vaginal dryness and dyspareunia and DIVA (Day-by-Day Impact of Vaginal Ageing) for subjective measures. Objective measures included VHI and VVHI (Vulvo-Vaginal Health Index). The follow-up was done at 4 and 8 weeks and at 3, 6, 9, 12, and 15 months. Multivariate analysis showed that the follow-up time was correlated with better VHI and VVHI (p<0.001). DIVA was also improved over time (p<0.001). All this implies that CO2 laser treatment of vulvovaginal atrophy significantly improves the symptoms in the long-lasting manner.

Similar results were obtained earlier by Salvatore S et al. [13], using CO2 laser treatment for 12 weeks in 50 women. This was a pilot study using the subjective VAS scale and objective Vaginal Health Index Score (VHIS) scale only. The results showed a statistically significant improvement in vaginal dryness, vaginal burning, vaginal itching, dyspareunia, and dysuria (p<0.001) at the 12-week follow-up. A recent study by Athanasiou S et al. [43] assessed the efficacy of microablative CO2 laser therapy in treating GSM in a follow-up period of 12 months using retrospective analysis at baseline and at 1, 3, 6, and 12 months after the last laser therapy. Of the 94 women included in the study, 35 were treated with 3 therapies, 35 with 4, and 24 with 5 laser therapies. All GSM statistical symptoms improved significantly. The results showed that 4-5 laser treatment might be superior in lowering the GSM symptoms than 3 therapies in short- as well as in long-term follow-up.

Gambacciani M et al. [44] introduced the Vaginal Erbium Laser Academy Study (VELAS) in 1500 postmenopausal women including eleven centers in Italy using the same protocol and the same Er:YAG laser technology. All centers used Female Sexual Function Index (FSFI), VAS, and VHI questionnaires for the evaluation of VEL on GSM symptoms. The first results of VELAS study [19] showed that VEL treatment significantly improves GSM at 12 months after the last laser application, whereas the effects decrease afterward. The study confirms that VEL is effective in the treatment of GSM with clinical effects similar to those exerted by the established local therapies.

Gaspar A et al. [45, 46] hypothesized that, by targeting the mucosal component of the urethral sphincter, urethral coaptation could be increased. In a pilot study, they assessed the possibility of a 4-mm intraurethral Er:YAG laser cannula in treating GSM in postmenopausal women (2 lasers sessions 3 weeks apart). The idea was to assess the new technology of using a laser to target the urinary mucosa for the relief of GSM symptoms. The thickness of the urethral mucosa and the vascularization of the submucosa are responsible for its sealing and therefore confer in the continence ability. By using the intraurethral procedure, the continence could be improved by enhancing the urethral tropism. After 3 months, dysuria improved in all the patients, frequency in 97% of patients, and urgency in 93% of patients. The VAS values for dysuria, urgency, and frequency decreased from baseline values 66, 58, and 49 to 8, 13, and 11 after 3 months and 20, 28, and 21 at a 6-month follow-up (p<0.0005). ICIQ-UI was also decreased from 13 at the baseline to 5.2 at a 3-month and to 8.1 at a 6-month follow-up (p<0.0005). As assessed by a questionnaire addressing QoL (ICIQ-UI SF) and the 1-hour pad test, therapeutic efficacy was measured at 3 and 6 months after the procedure. The results evaluated by ICIQ-SF questionnaire showed an improvement by 64% in average, at 3 months, and by 40%, at 6 months. A reduction of the quantity of leaked urine by 59% at 3 months and by 42% at 6 months was evaluated with the 1-hour pad test.

Bearing all these findings in mind we may conclude that intraurethral Er:YAG procedure could be the procedure of choice for the women with incontinence-dominated GSM. However, further randomized face-to-face Er:YAg procedures comparing a vaginal laser with intraurethral laser should be performed to confirm this basic idea.

## 8. Discussion

This is a nonsystematic review of the literature derived from PubMed database up to March 2019 (Table 1). Keywords “laser” and “urinary incontinence” yield 370 articles. After exclusion for incontinence in male, laser use in surgery, nonlaser techniques, and review articles, 24 papers met criteria regarding laser use in women with urinary incontinence. Laser effect in SUI patients was the primary goal in 21 studies, OAB in 1, GSM in 1, and histology in 1 study. A total of 1,452 patients were enrolled at the 1-36-month follow-up (mean 7.04 months). In average 1.9 laser sessions per patient, an average reduction in ICIQ-UI SF scores was 5.9, and an average 1-hour pad test reduction was 16 grams with an average continence rate after laser treatment of 44.5%.

Food and Drug Administration (FDA) position statement on fractional CO2 laser treatment claims that “although there are a number of indications enumerated for this technology, the specific indication for the treatment of vulvovaginal atrophy is not listed” [47] and recent 2018 FDA warns against the use of energy-based devices (EBDs), including laser and radiofrequency devices, to perform “vaginal rejuvenation” or vaginal cosmetic procedures [48]. International Urogynecology Association (IUGA) committee opinion [49] and International Continence Society (ICS) ICS/ISSVD best practice consensus document [50] states that “therapeutic advantages of nonsurgical laser-based devices in urogynecology can only be recommended after robust clinical trials have demonstrated their long-term complication profile, safety, and efficacy” and “at this point, laser is not recommended for routine treatment of the aforementioned conditions unless part of well-designed clinical trials or with special arrangements for clinical governance, consent, and audit,” respectively.

It is obvious that additional studies are needed to explore the long-term safety and efficacy of various laser therapies for genitourinary symptoms. However, a number of prospective observational studies show the effectiveness and safety of vaginal Er:YAG SMOOTH®, confirmed by randomized sham-controlled data [23]. Long-term studies demonstrate that the effects of Er:YAG SMOOTH® treatment are comparable to local hormone treatment [19]. Head to head study by Okui N. [51] showed that Er:YAG SMOOTH® therapy in women improved urinary incontinence as effectively as the tension-free vaginal tape (TVT) and transobturator tape (TOT) procedures. For patients with mixed urinary incontinence (MUI), some in the TVT and TOT groups showed exacerbation; however, all patients in the laser therapy group tended to improve.

## 9. Conclusion

“Laser” in medicine stands for a number of diverse devices. They radiate different energy at different wavelengths and produce different effects in different tissues.

At the moment there is no available published head to head study with different laser devices for SUI and GSM treatment.

However, long-term well designed prospective studies are still needed to disclose the effectiveness of laser and other EBDs in SUI and GSM treatment.

## Figures and Tables

**Table 1:** Clinical studies using different laser devices for the treatment of UI/GSM.

Author(s)	Laser type	Primary goal	Study type	N	Outcome	Follow-up (months)
Fistonic I, et al. [52] 2012	Er:YAG SMOOTH®	SUI	Obs	39	3.3^1^	6
Fistonic N, et al. [15] 2015	Er:YAG SMOOTH®	SUI	Obs	73	5^1^, 38.3^2^	6
Fistonic I, et al. [11] 2015	Er:YAG SMOOTH®	SUI	Obs	31	5.1^1^, 32.5^2^	6
Ogrinc BU, et al. [17] 2015	Er:YAG SMOOTH®	SUI/MUI	Obs	175	4.7^4^, 62^2^	12
Gambacciani M, et al. [19] 2015	Er:YAG SMOOTH®	SUI/VVA	Obs	19	6.4^1^, 11^3^,	6
Leshunov E, et al. [21] 2015	Er:YAG	SUI	Obs	37	5^3^	1
Khalafalla MM, et al. [53] 2015	Er:YAG SMOOTH®	SUI	Obs	50		6
Pardo J, et al. [20] 2016	Er:YAG SMOOTH®	SUI	Obs	42	8^1^, 38.1^2^	6
Tien YW, et al. [18] 2016	Er:YAG SMOOTH®	SUI/OAB	Obs	35	12^5^, 50^2^	6
Pitsouni E, et al. [25] 2016	CO2	GSM/SUI	Obs	35	4.7^1^	4
Perino A, et al.[31] 2016	CO2	OAB	Obs	30		1
Isaza GP, et al. [24] 2017	CO2	SUI	Obs	161	7.5^1^	36
Gaspar A, et al. [45] 2017	Er:YAG SMOOTH®	SUI	Obs	22	10^1^, 46^2^	6
Lin YH, et al. [34] 2017	Er:YAG SMOOTH®	SUI/OAB	Obs	30	4.5^1^	3
Lapii GA, et al. [10] 2017	Er:YAG SMOOTH®	SUI	Obs	98	Histology	2
Neimark AI, et al. [22] 2018	Er:YAG SMOOTH®	SUI	Obs	98	73^2^	2
Blaganje M, et al. [23] 2018	Er:YAG SMOOTH®	SUI	RCT	114	4^1^, 21^2^	3
Gaspar A, et al. [46] 2018	Er:YAG SMOOTH®	SUI	Obs	29	4.9^1^, 14^3^, 45^2^	6
Fistonic I, et al. [27] 2018	Er:YAG SMOOTH®	SUI	Obs	85	19^3^	6
Gambacciani M, et al. [19] 2018	Er:YAG SMOOTH®	SUI/GSM	Obs	114	4.2^1^	12
Okui N [51] 2018	Er:YAG SMOOTH®	SUI	Pro	50	11^1^, 31^3^	12
Pardo Shanz J, et al. [54] 2018	Er:YAG diode	SUI	Pro	19	8^1^, 26.3^2^	3
Samuels JB, et al. [55] 2019	CO2	GSM/SUI	Obs	25	65^2^	12
Lin YH, et al. [56] 2019	Er:YAG SMOOTH®	SUI	Obs	41	3.5^1^, 7.1^3^, 36.6^2^	6

SUI, Stress Urinary Incontinence; MUI, Mixed Urinary Incontinence; OAB, Overactive Bladder; VVA, Vulvo-Vaginal Atrophy; GSM, Genito-urinary Syndrome of Menopause.

ICIQ-UI, International Consultation on Incontinence Questionnaire-Urinary Incontinence Short Form; ISI, Incontinence Severity Index; KHQ, King's Health Questionnaire; PIFQ-7, Pelvic Floor Impact Questionnaire; OAB-Q SF, Overactive Bladder Questionnaire Short Form; Obs, Observational; RCT, randomized controlled trial; Pro, Prospective Outcomes.

1: Mean ICIQ score reduction; 2: percentage of the continent after follow-up; 3: mean pad test weight reduction (g); 4: mean ISI score reduction; 5: Mean KHQ (King's Health Questionnaire) score reduction.
